# Impact of symptom severity in patients with diarrhoea-predominant irritable bowel syndrome (IBS-D): results from two separate surveys of HCPs and patients with IBS-D

**DOI:** 10.1186/s12876-020-01252-9

**Published:** 2020-04-26

**Authors:** Anton Emmanuel, Richard William Goosey, Gwen Wiseman, Stephen Baker, Hans Törnblom

**Affiliations:** 1 University College Hospital, 235 Euston Road, London, NW1 2BU UK; 2 Kantar Health, Epsom, Surrey UK; 3 Former employee of Allergan plc, Marlow, Buckinghamshire UK; 4 Allergan Ltd, Marlow, Buckinghamshire UK; 5 Department of Internal Medicine & Clinical Nutrition, Institute of Medicine, Sahlgrenska Academy, University of Gothenburg, Gothenburg, Sweden

**Keywords:** Diarrhoea-predominant irritable bowel syndrome, Gastrointestinal symptom rating scale, IBS, Outcomes research, Patient-reported outcomes

## Abstract

**Background:**

Management of diarrhoea-predominant irritable bowel syndrome (IBS-D) is generally based on patient-reported symptoms; however, limited information on symptom severity exists. The objective of the study was to assess the impact of IBS-D severity on patient burden and patient and healthcare professional attitudes towards IBS.

**Methods:**

We conducted two web-based surveys of healthcare professionals and patients from Australia, Canada and Europe. We analysed patient characteristics and attitudes by IBS-D severity, which was assessed retrospectively using a composite of four variables: worst abdominal pain, IBS symptom frequency, Bristol Stool Form Scale and quality of life.

**Results:**

Of 679 healthcare professional respondents, one-third routinely classified patients by severity. The patient survey was completed by 513 patients with mild (26%), moderate (33%) and severe (41%) IBS-D, classified using the composite scale. Age, sex and treatment satisfaction did not change with severity; however, 19% of patients classified with severe IBS-D agreed with the statement: ‘When my IBS is bad, I wish I was dead’ versus 4 and 7% of patients with mild and moderate IBS-D, respectively (*p* < 0.05). Significantly more patients classified with severe IBS-D reported medication use versus mild IBS-D.

**Conclusion:**

Compared with milder symptoms, severe IBS-D was associated with increased medication use and a negative perspective of IBS-D. This highlights the need for a validated severity scale to inform treatment decisions.

## Background

Irritable bowel syndrome (IBS) is a chronic functional bowel disorder with a global prevalence of around 11% [1]. IBS is characterised by abdominal pain with altered bowel habits, such as a predominance of constipation (IBS-C), diarrhoea (IBS-D), or a mixed pattern of both (IBS-M), as well as urgency and bloating [2, 3]. Symptoms vary from mild to severe and intermittent to continuous, with incidence of mild IBS estimated to be ~ 40%, moderate ~ 35% and severe ~ 25%, based on patients’ self-perceived severity [4]. Patients who self-report severe IBS-D have been described as experiencing greater impairments in health-related quality of life (QoL), increased work and activity impairment and increased healthcare resource use compared to patients who self-report mild or moderate IBS-D [5–9].

To date, there is no established definition of severity for IBS. The Rome IV diagnostic criteria do not classify patients according to IBS severity, but set out a multifactorial approach for the diagnosis of IBS based on symptoms, primarily abdominal pain and diarrhoea [2]. The Rome IV criteria state that IBS treatment should be dependent upon symptom type and severity [2] (for example, linaclotide is recommended for patients with moderate-to-severe IBS-C [10]); however, no validated scale is suggested to assess this beyond those available for IBS as a whole, such as the Birmingham-IBS questionnaire, functional bowel disorder severity index and the IBS symptom severity scale (IBS-SSS). These scales do not take into account the multifactorial diagnostic approach set out by the Rome IV criteria [4, 11–14] and are not specific to IBS-D or IBS-C. Therefore, classification of IBS-D severity is dependent upon the type of scale used and whether the patient or physician makes the severity definition, as well as variables such as symptom intensity, time of assessment and degree of disability or impairment [15].

We conducted a study that surveyed (a) patients receiving treatment for IBS-D and (b) treating gastroenterologists and primary care physicians (PCPs) to assess the health burden of IBS-D on patients and the attitudes and perspectives of treating healthcare professionals (HCPs) towards IBS-D. Our primary analysis found that many patients are dissatisfied with their current treatment and feel under-supported by their HCPs, whilst the physicians themselves find IBS-D to be a challenging condition to manage [16]. One surprising finding of the study was that faecal urgency was reported as the most troublesome symptom, rather than the characteristic diarrhoea and abdominal pain.

The aim of this post-hoc subanalysis was to evaluate HCPs’ attitudes towards the classification of severity in IBS-D based on the survey data and to evaluate symptom burden, medication consumption and patients’ attitudes graded by severity, as defined using patient-reported variables.

## Methods

The study was comprised of two web-based, self-administered surveys of (a) patients with IBS-D and (b) treating HCPs. Detailed methodology of the surveys are described elsewhere [16]. Each structured questionnaire was administered via market research panels provided by Survey Sampling International and included individuals from Australia, Canada, France, Germany, Italy, Spain and the UK.

The 30-min patient survey was completed between January and February 2016. Patients with IBS-D opted in to complete the survey via an email link; they received a small monetary compensation for their time. The survey comprised 51 questions assessing patients’ attitudes towards their IBS and IBS treatments.

The 40-min HCP survey was completed between February and April 2016. Gastroenterologists and PCPs were included. HCPs were paid honoraria of up to $150 for participating in the survey. The analysis described here focusses on responses to questions around the assessment of IBS-D severity.

### Sample population

The screening criteria for inclusion have been described previously for both the patient and HCP surveys [16]. The HCP survey included HCPs who had consultations with patients with diagnosed IBS-D within 3 months prior to the survey, and had prescribed medication or recommended over-the-counter treatments for patients with IBS-D. The patient survey included patients with diagnosed IBS-D currently using medications for symptoms experienced within the preceding 12 months.

### Responses to statements

HCP and patient attitudes to statements were scored using a 7-point Likert scale (1 = completely disagree; 4 = neither agree nor disagree; 7 = completely agree). Participants who answered 6 or 7 were classed as agreeing with the statement; those who answered 1 or 2 were classed as disagreeing with the statement. Participants with a score of 3–5 were classed as neither agreeing nor disagreeing with the statement.

### Definition of IBS-D severity

For the HCP survey, definitions of severity (mild, moderate and severe) were provided to survey participants, as defined by the 2011 Rome Foundation Working Team report [4]. For more details, see Additional file 1.

In the patient survey, IBS-D severity was calculated using an algorithm comprising four variables chosen retrospectively to reflect the Rome IV diagnostic criteria [2], recent clinical trial data and clinical experience: worst abdominal pain (WAP) scored from 0 (no pain) to 10 (worst pain imaginable); frequency of IBS symptoms measured in days/months; stool consistency scored from Bristol Stool Form Scale (BSFS) 3 (stool is like a sausage but with cracks on its surface) to 7 (watery stool with no solid pieces, entirely liquid); and a measure of QoL assessed according to patient responses to the statement ‘Having IBS stops me enjoying life’, scored on a 7-point Likert scale. A symptom frequency score was created by taking the maximum number of days from four questions regarding average monthly frequency of stomach pain and/or diarrhoea symptoms. For the questions and response choices, see Additional file 1: Table S1.

**Fig. 1:**
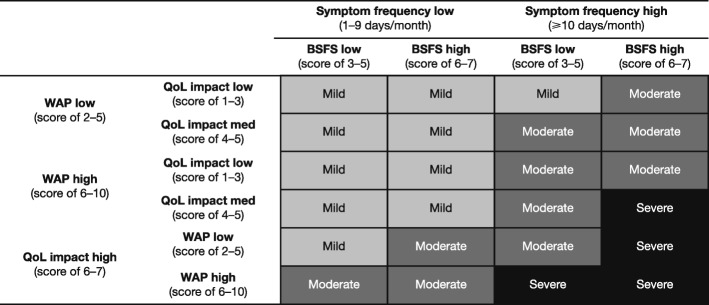
Criteria for the determination of IBS-D severity by WAP, frequency of symptoms, BSFS and QoL. Cut-offs for severity levels were arbitrary and based on clinical experience; frequency was based on the number of days with IBS symptoms. *BSFS* Bristol Stool Form Scale; *IBS* irritable bowel syndrome*; IBS-D* diarrhoea-predominant irritable bowel syndrome; *QoL* quality of life; *WAP* worst abdominal pain

Symptom frequency, WAP, BSFS and QoL scores were grouped into high or low categories (and medium for QoL), based on the judgement of the lead investigators and informed by prior experience in the therapeutic area. These categories were the basis of the algorithm used to stratify patients into groups of mild, moderate or severe IBS-D (Fig. 1). For a detailed description of the algorithm used to stratify patients by severity, see Additional file 1.

### Statistical analysis

Anonymised patient responses were analysed at the respondent record level and stratified by the mild/moderate/severe classification. Statistical analyses were performed on the differences between the three severity groups. For additional details regarding the statistical analyses, see Additional file 1.

## Results

### HCP survey

Of the 1460 HCPs screened, 313 gastroenterologists and 366 PCPs were eligible for inclusion and completed the survey. The demographics and caseloads of these HCPs are described elsewhere [16].

#### HCP classification of IBS-D severity

Approximately one-third each of PCPs (30%) and gastroenterologists (31%) reported that they routinely classify their IBS-D patients by severity (Table 1). Using the severity definitions provided, gastroenterologists estimated that 42% of their diagnosed or suspected IBS-D patients have mild, 41% have moderate and 18% have severe IBS-D. Similarly, PCPs estimated that 46, 40 and 14% of their diagnosed or suspected IBS-D patients have mild, moderate and severe IBS-D, respectively.

**Table 1:** Factors used by PCPs and gastroenterologists to classify patients with IBS-D by severity

HCPs, *N* (%)	PCPs (*n* = 366)	Gastroenterologists (*n* = 313)
Classifying patients by severity^a^	110 (30)	98 (31)
Classification based on:^b^		
Frequency/duration of symptoms	59 (54)	48 (49)
Impact on daily life	52 (47)	50 (51)
Type of symptoms	51 (46)	46 (47)
Intensity of symptoms	21 (19)	23 (23)
Abdominal pain intensity scale	19 (17)	21 (21)
Guidelines	3 (3)	8 (8)

^a^Based on responses to the question: ‘Do you classify or group your diagnosed IBS-D patients by severity in your day-to-day practice?’

^b^Based on responses to the question: ‘Please describe below how you classify or group your diagnosed IBS-D patients by severity in your day-to-day practice’ (select all that apply), expressed as a proportion of those HCPs answering ‘yes’ to the previous question

*HCP* healthcare professional; *IBS-D* diarrhoea-predominant irritable bowel syndrome; *PCP* primary care physician

Very few of the HCPs who routinely assessed IBS-D severity reported using guidelines to make this classification (3 and 8% of PCPs and gastroenterologists, respectively). In general, around half of the HCPs who classified patients by IBS-D severity reported that this was based on the frequency/duration of symptoms, type of symptom and/or impact on daily life (Table 1). Of those HCPs who did not routinely classify their patients by severity in their day-to-day practice, impact on QoL, level of abdominal pain, number of symptomatic days in an average month and frequency of diarrhoea were the factors most likely to be used for this purpose; gastroenterologists were more likely to use frequency of diarrhoea, whether a patient responds to treatment and stool consistency to make a severity assessment (Additional file 1: Fig. S1).

**Fig. 2:**
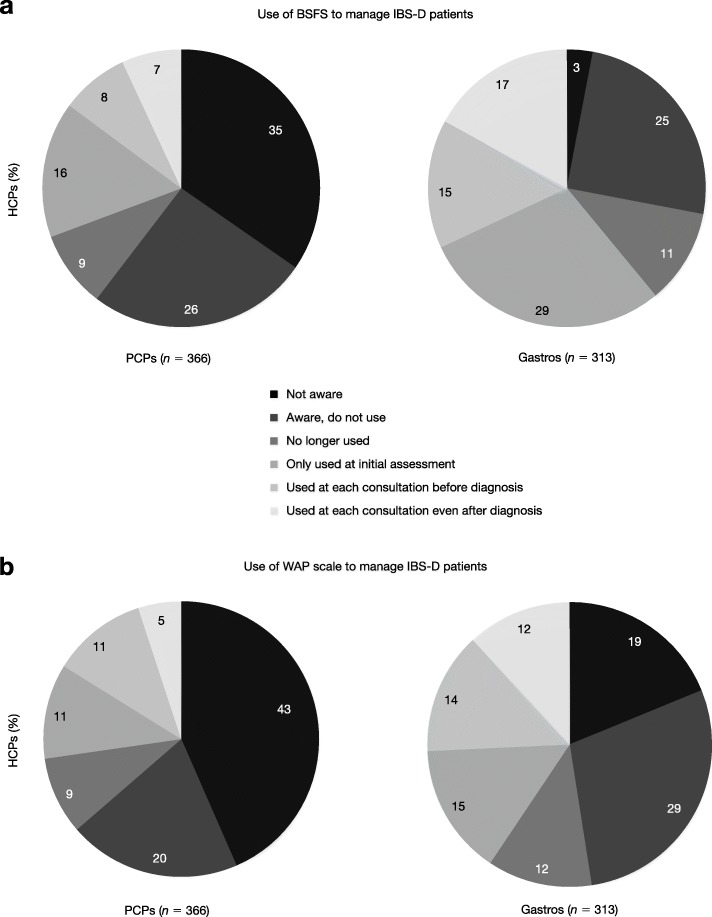
Use of the BSFS and WAP scale in the management of patients with IBS-D. Based on responses to the question: ‘At what point, if at all, do you use the following scales in managing your IBS-D patients?’ (**a**) *p* < 0.05 for PCPs vs gastroenterologists unaware of the BSFS, only using the BSFS at initial assessment with patient, and using the BSFS at each consultation with patient before and after diagnosis. (**b**) *p* < 0.05 for PCPs vs gastroenterologists unaware of the WAP scale, aware of the WAP scale but not using, and using the WAP scale at each consultation with patient before and after diagnosis. *BSFS* Bristol Stool Form Scale; *Gastro* gastroenterologist; *HCP* healthcare professional; *IBS-D* diarrhoea-predominant irritable bowel syndrome; *PCP* primary care physician; *WAP* worst abdominal pain

Use and awareness of the BSFS and WAP scale varied widely between the groups of HCPs: 61 and 63% of PCPs reported a lack of awareness/use of the BSFS and WAP scale, respectively, compared to 28 and 48% of gastroenterologists, respectively (Fig. 2).

### Patient survey

#### Demographics and health characteristics

Overall, 8627 patients were screened, of whom 513 were eligible and completed the survey. The mean age was 40.9 years and 70% were female [16]. All 513 patient responses were anonymised and assessed for severity: 193 patients (38%) had severe IBS-D, 158 (31%) had moderate IBS-D and 124 (24%) had mild IBS-D (Table 2). In total, 38 patients (7%) did not meet the severity criteria and were excluded from the severity analysis. A significantly greater proportion of patients with severe IBS-D reported depression and fibromyalgia, and had undergone several prior diagnostic tests compared to patients with mild or moderate IBS-D (Table 2). Age and sex were not associated with IBS-D severity.

**Table 2:** Demographics and health characteristics by IBS-D severity

	Mild IBS-D (*n* = 124)	Moderate IBS-D (*n* = 158)	Severe IBS-D (*n* = 193)
Female, *N* (%)	81 (65)	109 (69)	143 (74)
Mean age, years (SD)	40.4 (10.9)	40.5 (11.2)	42.0 (12.1)
Most common comorbidities, *N* (%)^a, b^			
Anxiety	42 (34)	60 (38)	73 (38)
Depression	26 (21)	35 (22)	65 (34)*^†^
Migraine	31 (25)	41 (26)	55 (28)
Gastric reflux	23 (19)	38 (24)	39 (20)
Lactose intolerance	11 (9)	23 (15)	30 (16)
Fibromyalgia	3 (2)	5 (3)	22 (11)*^†^
Diarrhoea due to bacterial infection	13 (10)	9 (6)	10 (5)
Diagnostic testing history, *N* (%)^c, d^			
Blood tests	94 (76)	121 (77)	158 (82)
Stool test	72 (58)	95 (60)	128 (66)
Endoscopy/colonoscopy	59 (48)	91 (58)	134 (69)*^†^
Food allergy tests	42 (34)	56 (35)	88 (46)

*p < 0.05 vs patients with mild IBS-D; ^†^*p* < 0.05 vs patients with moderate IBS-D.

^a^Based on responses to the question: ‘Which of the following conditions, if any, have you been diagnosed with by a doctor?’

^b^Reported in ≥10% of patients

^c^Based on responses to the question: ‘Which of the following tests have been carried out since you first experienced symptoms of IBS?’

^d^Reported in ≥20% of patients

*IBS* irritable bowel syndrome; *IBS-D* diarrhoea-predominant irritable bowel syndrome; *SD* standard deviation

#### IBS symptom characteristics

The mean duration of IBS symptoms was 9.8 years, without any significant difference in duration across severity groups. The most common reason overall for a first visit to an HCP was a large impact on QoL for all severity groups (Additional file 1: Table S2).

**Fig. 3:**
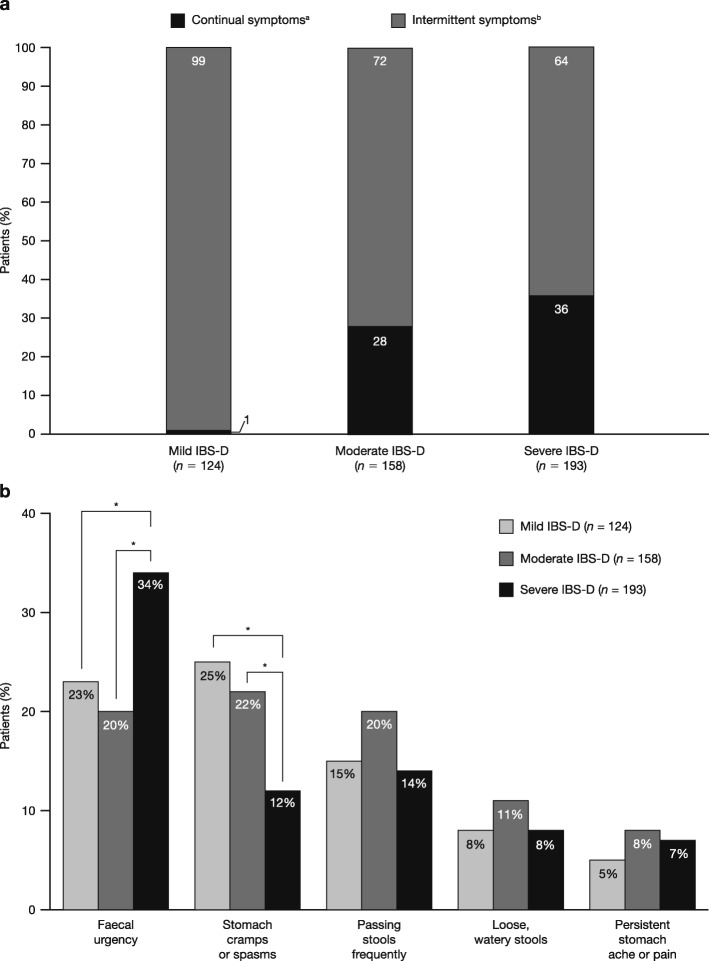
Symptom patterns and most troublesome symptoms by IBS-D severity. (**a**) Symptom patterns over the past 3 months by IBS-D severity. *p* < 0.05 for all comparisons (mild vs severe and moderate vs severe; continual and intermittent). Based on responses to the question: ‘Which best describes the pattern of your IBS symptoms over the past 3 months?’ ^a^Defined as experiencing some IBS symptoms every day. ^b^Defined as having some days without any IBS symptoms. (**b**) Most troublesome symptoms currently experienced by IBS-D severity. Based on the first selected response to the question: ‘Which of the symptoms you currently experience trouble you the most?’ (bloating was not included as a potential response). *p < 0.05. *IBS* irritable bowel syndrome*; IBS-D* diarrhoea-predominant irritable bowel syndrome

A greater proportion of patients with severe IBS-D reported continual symptoms over the 3 months prior to the survey, compared to those with mild or moderate IBS-D (*p* < 0.05 for both comparisons; Fig. 3a). For individual IBS symptoms, a larger proportion of patients with severe IBS-D listed urgency as common, compared to patients with mild or moderate IBS-D (47% vs 35% each for mild and moderate; *p* < 0.05 for both comparisons) and a larger proportion with severe IBS-D listed faecal incontinence as common, compared to mild or moderate IBS-D (22% vs 16 and 13%, respectively; *p* < 0.05 for severe vs moderate groups; Additional file 1: Table S2). Finally, patients with severe IBS-D were more likely to report faecal urgency as the most troublesome symptom, compared to patients with mild or moderate IBS-D (34% vs 23 and 20%, respectively; *p* < 0.05 each; Fig. 3b).

#### Medication use

Patients with severe IBS-D were more likely to use antidiarrhoeals or antidepressants compared to patients with mild or moderate IBS-D. For additional information regarding medication use, see Table 3. Treatment satisfaction for all medications considered was not found to be associated with IBS-D severity (data not shown).

**Table 3:** Medication use by IBS-D severity

*N* (%)	Mild IBS-D (*n* = 124)	Moderate IBS-D (*n* = 158)	Severe IBS-D (*n* = 193)
Types of medication used over the past 12 months^a^			
Antidiarrhoeal	94 (76)	124 (78)	167 (87)*^†^
Antispasmodic	71 (57)	99 (63)	112 (58)
Analgesic	17 (14)	27 (17)	33 (17)
Codeine-based painkiller	18 (15)	28 (18)	43 (22)
Antidepressant	14 (11)	25 (16)	38 (20)*
Other	9 (7)	11 (7)	15 (8)
Current medication use^b^			
OTC medication only	59 (48)	66 (42)	75 (39)
Prescription and OTC medication	30 (24)	46 (29)	69 (36)*
Prescription medication only	35 (28)	46 (29)	49 (25)

*p < 0.05 vs patients with mild IBS-D; ^†^*p* < 0.05 vs patients with moderate IBS-D.

^a^Based on responses to the question: ‘Which of the following have you taken in the past 12 months for your IBS?’

^b^Based on responses to the question: ‘Do you take either of the following to help manage your IBS?’

*IBS* irritable bowel syndrome; *IBS-D* diarrhoea-predominant irritable bowel syndrome; *OTC* over-the-counter

#### Patient attitudes

**Fig. 4:**
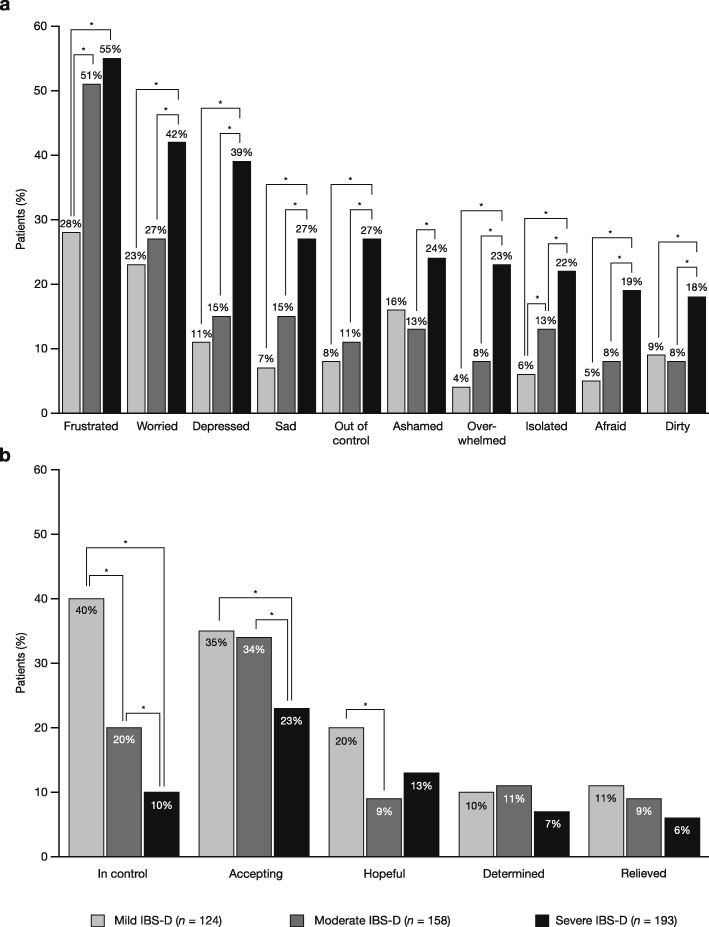
Patients’ feelings about having IBS by IBS-D severity. Based on responses to the question: ‘How do you currently feel about having IBS?’ (**a**) Negative emotions associated with IBS. (**b**) Positive emotions associated with IBS. *p < 0.05. *IBS* irritable bowel syndrome; *IBS-D* diarrhoea-predominant irritable bowel syndrome

Overall patient attitudes towards IBS-D are described in the initial overall report [16]. Response frequencies on negative emotions were significantly higher in patients with severe IBS-D for all emotions, compared to mild or moderate IBS-D, whereas response frequencies on positive emotions such as feeling ‘in control’ or ‘accepting’ were generally lower for patients with severe IBS-D compared to mild or moderate IBS-D (10% vs 40 and 20%, respectively, for ‘in control’; 23% vs 35 and 34%, respectively, for ‘accepting’; Fig. 4).

Compared to patients with mild or moderate IBS-D, there was a tendency for a significantly greater proportion of patients with severe IBS-D to report agreement with negative statements related to their IBS; over half (55%) of patients with severe IBS-D were constantly worried about when their symptoms would return, compared to patients with mild or moderate IBS-D (19 and 30%, respectively; *p* < 0.05 each). Further, 19% of patients with severe IBS-D agreed with the statement: ‘When my IBS is bad, I wish I was dead’, compared to 4% of patients with mild IBS-D and 7% of patients with moderate IBS-D (*p* < 0.05 for both comparisons; Additional file 1: Fig. S2a).

Similar severity associations were observed in terms of patients’ attitudes towards HCPs and services (Additional file 1: Fig. S2b). A significantly greater proportion of patients with severe IBS-D agreed with statements that there should be more services and education available for patients with IBS, compared to patients with mild or moderate IBS-D (59% vs 27 and 35%, respectively; *p* < 0.05 each).

Regarding current therapies, 62% of patients with severe IBS-D agreed with the statement: ‘I would use a daily treatment for the rest of my life if it prevented IBS symptoms’, compared to 26% of patients with mild IBS-D (*p* < 0.05) and 49% of patients with moderate IBS-D (*p* < 0.05; Additional file 1: Fig. S2c).

## Discussion

This post-hoc analysis indicates that increasing severity of IBS-D was associated with increased medication use and a negative impact on patients’ attitudes towards the condition itself, as well as HCPs, services and available treatments. In addition, we found that the majority of HCPs surveyed did not routinely assess severity in their patients with IBS. There is therefore a need for a standardised, multidimensional scale to assess severity in IBS-D, including measures of self-reported outcomes covering health-related QoL, psychosocial factors and burden of illness associated with IBS-D, particularly as new treatments emerge that are specific for this condition. A more complete understanding of symptom severity could not only improve the management and treatment of IBS-D, but could also inform patient stratification during future clinical trials to assess efficacy and safety across severity subgroups. One available method that could assist clinicians in monitoring symptoms is the IBS-D Daily Symptom Diary and Event Log, a patient-reported outcome measure designed to gauge treatment benefit, which includes measures of patient impression of severity and change over time [17–19].

In those HCPs who did routinely assess severity, this was largely based on the frequency/duration of symptoms, type of symptom and perceived impact on patients’ daily lives. It is particularly noteworthy, given that most IBS patients are managed in the community, that a large proportion of PCPs were found to be unaware of the BSFS and WAP scale. Among PCPs and gastroenterologists, only half of those surveyed used either scale in their clinical practice, despite the inclusion of the BSFS in the Rome IV criteria [2]. Further, very few respondents indicated that they used guidelines in the assessment of severity, suggesting that current IBS-D guidelines are inadequate in this respect. While various scales for the assessment of severity in IBS have been reported previously, such as the Birmingham-IBS questionnaire and IBS-SSS, these assessments are not specific to IBS-D [11–13].

In the current analysis, we used a composite severity scale to define subgroups of patients with IBS-D. This algorithm, although limited by the data already collected in the primary survey, was retrospectively designed to reflect the latest Rome IV diagnostic criteria [2], capturing information related to four key variables (abdominal pain, frequency of IBS symptoms, stool form and QoL). As such, this work was not powered to present a definitive research-oriented severity index, but rather to assess differences in physical symptoms, attitudes and behaviours in order to inform clinicians’ future management strategies according to IBS severity. A prospectively developed severity index for IBS-D will require external validation for use in clinical practice [20, 21]. This validation should include validation of the content, construct and criteria, as well as an assessment of the reliability and responsiveness of the index, with consideration given to the target sample numbers required a priori [22, 23].

IBS-D imposes a substantial burden on patients with the condition, who can experience troublesome symptoms, such as urgency, for long periods of time. Patients also express high levels of dissatisfaction with available treatments, which demonstrates an unmet need for the satisfactory management of these patients. This is important to note, as patient education has been shown to reduce IBS symptom severity [24, 25] and to improve QoL [25], suggesting that management of patients’ attitudes towards IBS will also help them manage their symptoms. Indeed, we found that a greater proportion of patients classified with severe IBS-D agreed that more education should be available.

Rome IV criteria state that IBS treatment should be dependent upon symptom type and severity, with initial treatment involving reassurance and diet and lifestyle modifications [2]. Other treatment options include opioid antagonists, bile acid sequestrants, probiotics, antibiotics, and 5-HT_3_ antagonists, yet prescription medications appear to be underutilised [26]. We observed in this study that while the majority of patients had received antidiarrhoeals, around 40% of patients with severe IBS-D were not taking any prescription medications, instead relying on over-the-counter medications, despite over 60% of patients with severe IBS-D expressing a willingness to receive regular treatment.

These results should be interpreted in light of the study limitations. The survey respondents were limited to those residing in seven countries, with only five countries (France, Germany, Italy, Spain and the UK) representing Europe, and attitudes to IBS-D severity may be subject to intercountry variation. Participants in the survey self-identified as having been diagnosed with IBS-D by an HCP, which may have led to selection bias, as it is possible that some patients may have under or overestimated the frequency and severity of their symptoms. In the present study, IBS patients made up a greater proportion of the PCP caseload than has been previously reported, which may be due to a greater prevalence of IBS-D, an increase in HCP visits for IBS or a sample selection bias [16]. As 60% of patients were required to have previously seen a gastroenterologist for their IBS-D, this patient population may have been skewed, leading to a higher estimate of patients suffering from severe IBS-D symptoms. Likewise, the requirement for gastroenterologists to be consultant grade may have excluded the perspectives of younger HCPs. Other limitations of the study were that a large proportion of the responses to the surveys were neutral and that there is a potential for bias towards the most ‘agreeable’ answer, which may also be affected by the response order [16]. In addition, the limitations of the survey itself include the use of a single question to assess troublesome symptoms (patients may have several highly troublesome symptoms), and also the lack of appropriate response options for certain questions. Indeed, a proportion of patients with severe IBS-D selected their most troublesome symptom as ‘other’ (ie not listed in this questionnaire). In particular, the troublesome symptom question did not take into account the severity of individual symptoms. Further, there may be inherent biases introduced by the use of the Likert format, such as acquiesce bias (the tendency for respondents to agree with statements) [27]. Finally, there are limitations to the severity algorithm used in this analysis. Although the categories selected to assess severity (WAP, BSFS, symptom frequency and QoL) reflected the key factors used to assess IBS-D in the clinic, the depth of available information, particularly within the QoL and symptom frequency categories, was limited by the retrospective development of the severity scale. This scale does not assess certain factors reported as important drivers of reduced QoL, such as abdominal distension or urgency [28]. However, this is also true of the IBS-SSS, which does not assess urgency and incontinence. That being said, this scale was able to demonstrate some clear differences in patient characteristics and attitudes according to the level of IBS-D severity, and therefore highlights the need for a validated scale.

## Conclusions

This post-hoc analysis demonstrated that patient characteristics and attitudes differ substantially according to the severity of their IBS-D symptoms. This indicates a need for the development of a symptom severity index. Further attention by the Rome IV Committees is warranted as part of their multiaxial work-up of patients with functional disorders [4]. We also identified a distinct need for improved pharmacological and supportive management of patients with IBS-D in order to reduce symptom burden, particularly in those with more severe IBS-D.

## Supplementary information

**Additional file 1:** Methods and materials. **Table S1.** Questions and responses used in the calculation of IBS-D severity. **Table S2.** Symptom burden and reason for first HCP visit by IBS-D severity. **Fig. S1.** Symptom categories used by HCPs to classify IBS-D severity. Based on responses to the question: ‘Which, if any, of the following would you use to classify severity of IBS-D in your day-to-day practice?’ Respondents limited to those HCPs who answered ‘no’ to the question: ‘Do you classify or group your diagnosed IBS-D patients by severity in your day-to-day practice?’ **Fig. S2.** Patient-reported perspectives on IBS by severity. Patient attitudes towards statements on (a) IBS-D, (b) HCPs and services, and (c) current therapies and treatment goals.

## Data Availability

The datasets generated and or analysed during the current study are not publicly available as they were used under license for the current study, but are available from the corresponding author upon reasonable request, with the permission of Allergan plc.

## Abbreviations

BSFS: Bristol Stool Form Scale
HCP: Healthcare professional
IBS: Irritable bowel syndrome
IBS-C: Constipation-predominant irritable bowel syndrome
IBS-D: Diarrhoea-predominant irritable bowel syndrome
IBS-M: Mixed subtype irritable bowel syndrome
IBS-SSS: IBS symptom severity scale
PCP: Primary care physician
QOL: Quality of life
WAP: Worst abdominal pain
