# Supplementary material for: Impact of symptom severity in patients with diarrhoea-predominant irritable bowel syndrome (IBS-D): results from two separate surveys of HCPs and patients with IBS-D
Source: BMC Gastroenterol. 2020 Apr 26;20:127. doi: 10.1186/s12876-020-01252-9 (PMC7183708; doi:10.1186/s12876-020-01252-9)

**Additional file 1. Methods and materials**

**Definition of IBS-D severity**

For the healthcare professional (HCP) survey, mild diarrhoea-predominant irritable bowel syndrome (IBS-D) was defined as mild or intermittent abdominal pain, little or no psychological distress, with occasional impact on activities. Moderate IBS-D was defined as moderate or frequent abdominal pain, more psychological distress, with more frequent impact on activities. Severe IBS-D was defined as very frequent or continuous abdominal pain, high psychological distress, with frequent or constant impact on activities. The survey also captured the criteria that HCPs were currently using to assess IBS-D symptom severity.

For the patient survey, all 513 patient responses were included and anonymised. Data were analysed at the respondent record level with arbitrary cut-offs based on judgement informed by prior experience in the therapeutic area. Responses to the questions on the four variables were grouped as described below. Responses to questions on worst abdominal pain (WAP) score were grouped into high (answer = 6–10) and low (answer = 1–5). An average monthly symptom frequency variable was created by taking the maximum number of days from four questions on average monthly frequency of stomach pain and frequency of diarrhoea. Respondents answering ‘continual symptoms’ when asked about the pattern of their IBS symptoms were given a frequency value of 30 days per month. Frequency variables were grouped into high (≥ 10 days/month) and low (1–9 days/month). Responses to questions on Bristol Stool Form Scale (BSFS) score were grouped into high (answer = 6–7) and low scores (answer = 3–5). Responses to the question regarding the impact of IBS on the patient’s enjoyment of life (quality of life [QoL]) were grouped into high impact (answer = 6–7), medium impact (answer = 4–5) and low impact (answer = 1–3). Composite data of these four criteria meant patients could be stratified into groups of mild, moderate or severe IBS-D as per Fig. 1. The basis of the classification is as follows: for severe IBS-D, there must be a high frequency of symptoms and a high impact on QoL, plus high BSFS and/or high WAP. If the BSFS, WAP and symptom frequency were all high, individuals reporting only a medium impact on their QoL would also classify as having severe IBS-D. To classify with moderate IBS-D, the criteria for severe IBS-D must not be met in addition to having any two of the four variables classified as high. If both BSFS and WAP were high, then additional high impact on QoL or high frequency of symptoms would also be classified as moderate. For mild IBS-D, there must be a low frequency of symptoms and low or medium impact on QoL. If both BSFS and WAP were low, the classification would be mild even if there was a high frequency of symptoms or high impact on QoL. The full data set was stratified by the above mild/moderate/severe classifications and statistical analyses were performed on the differences between the three groups.

As previously stated, the cut-offs for the classification of severity described above were based on judgement informed by prior experience in the therapeutic area, and were implemented to analyse the available patient survey data.

**Statistical analysis**

Two-tailed *t*-tests were performed for the means and proportions from independent groups (mild, moderate, severe IBS-D; HCPs and primary care physicians) to compare demographics, characteristics and attitudes data at a 5% risk level, with *p* < 0.05 denoting significance. Analyses were completed in Microsoft Excel (Microsoft, Redmond, WA, USA).

**Additional file 1: Table S1** Questions and responses used in the calculation of IBS-D severity

|  | **Question** | **Response selections** |
| --- | --- | --- |
| Worst abdominal pain | In the past 3 months when you have experienced your irritable bowel syndrome symptoms, please indicate the worst abdominal pain you typically feel on a scale of 0 to 10, where 0 indicates no pain and 10 the worst pain imaginable. | *Please select one answer* 0 1 2 3 4 5 6 7 8 9 10 |
| Symptom frequency | Which best describes the pattern of your irritable bowel syndrome symptoms over the past 3 months? | *Tick one only^a^*   1. **Continual symptoms**: You experienced some irritable bowel syndrome symptoms every day 2. **Intermittent symptoms**: You had some days without any irritable bowel syndrome symptoms |
| Symptom frequency | In the past 3 months, how many days per month, on average, have you experienced each of your irritable bowel syndrome symptoms? | *Please enter a whole number of days for each symptom^b^*   - Loose watery stools - Passing stools frequently - Stomach cramps or spasms - Persistent stomach ache or pain   Other symptoms included in the survey, but not incorporated into the IBS-D symptom frequency element of severity assessment: flatulence*,* faecal urgency*,* faecal incontinence, a sense of incomplete evacuation*,* passing mucus, nausea*,* fatigue/lack of energy |
| Stool consistency (BSFS) | In the past 3 months, when you have experienced your irritable bowel syndrome symptoms, which of the following best describes your typical stool consistency? | *Please select one answer*   - Like a sausage but with cracks on its surface (eg BSFS 3) - Like a sausage or snake, smooth and soft - Soft blobs with clear cut edges (passed easily) - Fluffy pieces with ragged edges, a mushy stool - Water, no solid pieces, entirely liquid (eg BSFS 7) - Other (please specify) - I would rather not say |
| Quality of life | We would now like to find out more about you as a person and how you view irritable bowel syndrome. We wish to understand the extent to which you agree or disagree with a number of statements. | *Please answer using a scale from 1 to 7, where 1 means completely disagree, 4 means neither agree nor disagree and 7 means completely agree*   - Having irritable bowel syndrome stops me enjoying life |

^a^If the respondents answered ‘continual symptoms’ when asked about the pattern of their IBS symptoms, they were given a frequency score of 30 days per month.
^b^The maximum number of days provided for any of the four symptoms was used as the symptom frequency score. Scores for patients with intermittent symptoms were grouped into high (≥ 10 days/month) and low (1–9 days/month).
*BSFS* Bristol Stool Form Scale; *IBS-D* diarrhoea-predominant irritable bowel syndrome.

**Additional file 1: Table S2** Symptom burden and reason for first HCP visit by IBS-D severity

|  | **Mild IBS-D (*n* = 124)** | **Moderate IBS-D (*n* = 158)** | **Severe IBS-D (*n* = 193)** |
| --- | --- | --- | --- |
| Mean length of time with symptoms, years (SD)^a^ | 8.7 (8.6) | 9.7 (9.4) | 10.7 (10.1) |
| Reason for first HCP visit, *N* (%)^b,c^ |  |  |  |
| Increasing frequency of my symptoms | 49 (40) | 71 (45) | 108 (56)*^†^ |
| The condition was having a large impact on my quality of life | 59 (48) | 70 (44) | 105 (54) |
| Increasing severity of my symptoms | 46 (37) | 60 (38) | 94 (49)*^†^ |
| I was worried about the length of time I’d had my symptoms for | 50 (40) | 76 (48) | 89 (46) |
| Fear of serious or life-threatening condition | 29 (23) | 28 (18) | 56 (29)^†^ |
| Treatments I tried myself did not relieve the symptoms | 26 (21) | 36 (23) | 54 (28) |
| A friend or family member advised me to go to an HCP | 27 (22) | 28 (18) | 49 (25) |
| I read information on the internet that made me suspect it was IBS | 21 (17) | 43 (27)* | 43 (22) |
| Family history of cancer (eg bowel or stomach) | 9 (7) | 14 (9) | 25 (13) |
| I experienced new/different symptoms | 11 (9) | 13 (8) | 19 (10) |
| I read information in magazines/newspapers that made me suspect it was IBS | 4 (3) | 10 (6) | 19 (10)* |
| Most common symptoms before treatment, *N* (%)^d,e^ | | | |
| Diarrhoea | 124 (100) | 158 (100) | 193 (100) |
| Stomach pain or discomfort | 88 (71) | 121 (77) | 158 (82)* |
| Stomach cramps or spasm | 88 (71) | 111 (70) | 139 (72) |
| Bloating | 62 (50) | 88 (56) | 104 (54) |
| Urgency | 43 (35) | 56 (35) | 90 (47)*^†^ |
| Mucus | 25 (20) | 27 (17) | 42 (22) |
| Faecal incontinence | 20 (16) | 20 (13) | 42 (22)^†^ |

**p* < 0.05 vs patients with mild IBS-D; ^†^*p* < 0.05 vs patients with moderate IBS-D.
^a^Based on responses to the question: ‘For how long have you experienced your IBS symptoms?’
^b^Based on responses to the question: ‘What made you decide to arrange your first appointment with a healthcare professional about the symptoms of IBS you were experiencing?’
^c^Reported in ≥ 10% of patients in any group.
^d^Based on responses to the question: ‘Which, if any, of the following symptoms does your IBS include before you take any treatment, if you take any?’
^e^Reported in ≥ 10% of patients in any group.
*HCP* healthcare professional; *IBS* irritable bowel syndrome; *IBS-D* diarrhoea-predominant irritable bowel syndrome; *SD* standard deviation.


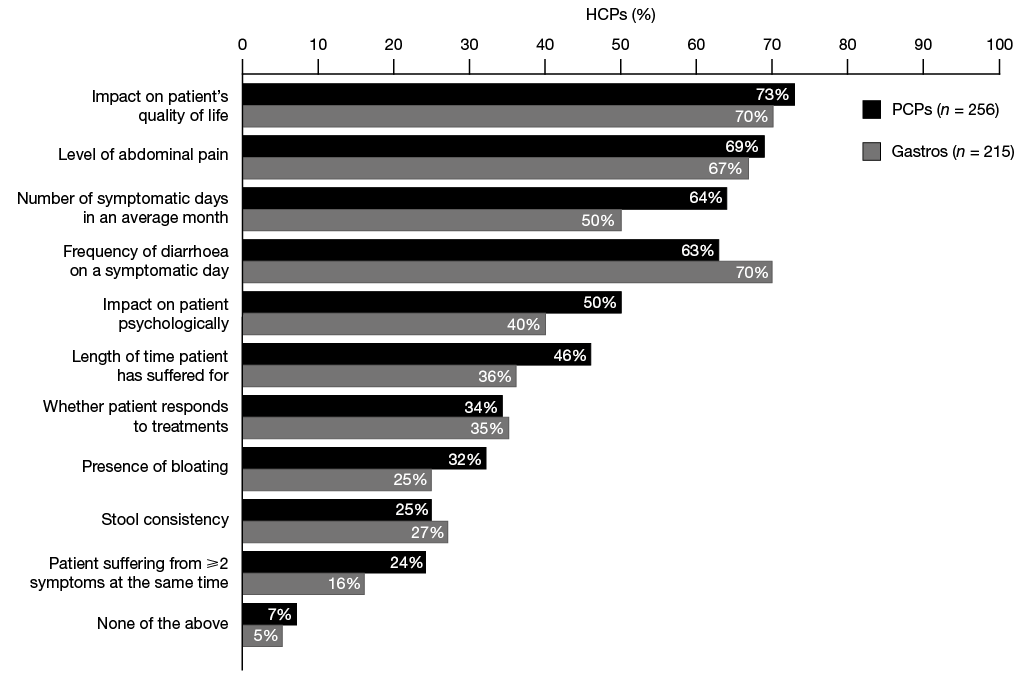
**Additional file 1: Fig. S1** Symptom categories used by HCPs to classify IBS-D severity. Based on responses to the question: ‘Which, if any, of the following would you use to classify severity of IBS-D in your day-to-day practice?’ Respondents limited to those HCPs who answered ‘no’ to the question: ‘Do you classify or group your diagnosed IBS-D patients by severity in your day-to-day practice?’ *Gastro* gastroenterologist; *HCP* healthcare professional; *IBS-D* diarrhoea-predominant irritable bowel syndrome; *PCP* primary care physician.

**Additional file 1: Fig. S2** Patient-reported perspectives on IBS by severity. Patient attitudes towards statements on (**a**) IBS-D, (**b**) HCPs and services, and (**c**) current therapies and treatment goals. *HCP* healthcare professional; *IBS* irritable bowel syndrome; *IBS‑D* diarrhoea-predominant irritable bowel syndrome.


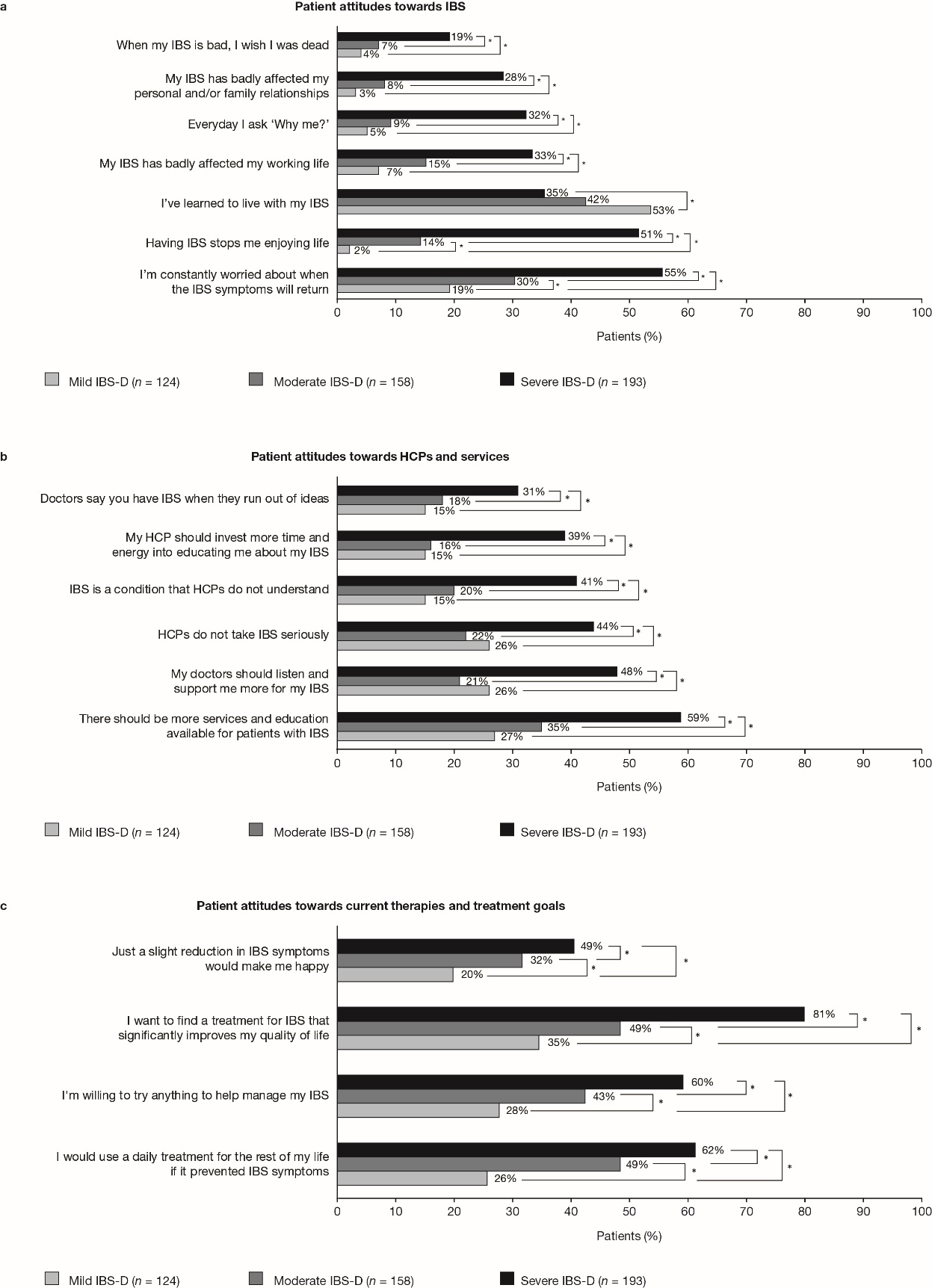

Supplement: Supplementary file 1 — Additional file 1: Methods and materials. Table S1. Questions and responses used in the calculation of IBS-D severity. Table S2. Symptom burden and reason for first HCP visit by IBS-D severity. Fig. S1. Symptom categories used by HCPs to classify IBS-D severity. Based on responses to the question: ‘Which, if any, of the following would you use to classify severity of IBS-D in your day-to-day practice?’ Respondents limited to those HCPs who answered ‘no’ to the question: ‘Do you classify or group your diagnosed IBS-D patients by severity in your day-to-day practice?’ Fig. S2. Patient-reported perspectives on IBS by severity. Patient attitudes towards statements on (a) IBS-D, (b) HCPs and services, and (c) current therapies and treatment goals. [file 12876_2020_1252_MOESM1_ESM.docx]
